# TRAIL/DR5 Signaling Promotes Macrophage Foam Cell Formation by Modulating Scavenger Receptor Expression

**DOI:** 10.1371/journal.pone.0087059

**Published:** 2014-01-22

**Authors:** Fang Fang Liu, Xiao Wu, Yun Zhang, Yan Wang, Fan Jiang

**Affiliations:** 1 Key Laboratory of Cardiovascular Remodeling and Function Research, Qilu Hospital, Shandong University, Jinan, Shandong Province, China; 2 Department of Cardiology, Beijing Hospital, Beijing, China; The University of Texas MD Anderson Cancer Center, United States of America

## Abstract

Tumor necrosis factor-related apoptosis-inducing ligand (TRAIL/Apo2L) has been shown to have protective effects against atherosclerosis. However, whether TRAIL has any effects on expression of macrophage scavenger receptors and lipid uptake has not yet been studied. Macrophage lines RAW264.7 and THP-1, and mouse primary peritoneal macrophages, were cultured in vitro and treated with recombinant human TRAIL. Real-time PCR and western blot were performed to measure mRNA and protein expressions. Foam cell formation was assessed by internalization of acetylated and oxidized low-density lipoproteins (LDL). Apoptosis was measured by terminal deoxynucleotidyl transferase-mediated dUTP nick end labeling. We found that TRAIL treatment increased expression of scavenger receptor (SR)-AI and SR-BI in a time- and dose-dependent manner, and this effect was accompanied by increased foam cell formation. These effects of TRAIL were abolished by a TRAIL neutralizing antibody or in DR5 receptor-deficient macrophages. The increased LDL uptake by TRAIL was blocked by SR-AI gene silencing or the SR-AI inhibitor poly(I:C), while SR-BI blockade with BLT-1 had no effect. TRAIL-induced SR-AI expression was blocked by the inhibitor of p38 mitogen-activated protein kinase, but not by inhibitors of ERK1/2 or JNK. TRAIL also induced apoptosis in macrophages. In contrast to macrophages, TRAIL showed little effects on SR expression or apoptosis in vascular smooth muscle cells. In conclusion, our results demonstrate that TRAIL promotes macrophage lipid uptake via SR-AI upregulation through activation of the p38 pathway.

## Introduction

Tumor necrosis factor (TNF)-related apoptosis-inducing ligand (TRAIL), also known as Apo-2 ligand (Apo-2L) or TNFSF10, is a member of the TNF super family of cytokines [1]–[3]. The primary biological action of TRAIL is induction of apoptosis in transformed tumor cells [1]. TRAIL induces cell apoptosis via its receptors TRAIL-R1 (also known as DR4) or TRAIL-R2 (DR5) (mouse has the DR5 gene only), which are type I trans-membrane proteins containing a cytoplasmic death domain [2]–[4]. TRAIL ligation to DR4/5 receptors causes recruitment of the adaptor molecule FADD and apoptosis initiators caspase-8 and −10, forming a primary signaling complex called death-inducing signaling complex (DISC) [1], [2], [4]. Alternatively, TRAIL may also trigger formation of a secondary signaling complex containing FADD, caspase-8, RIP1, TRAF2, TRADD and NEMO, in which the ligand and cognate receptor are absent. This signaling route activates nuclear factor (NF)-κB and mitogen-activated protein kinases (MAPKs) such as JNK and p38, and is thought to have a pro-survival role [1], [2], [4].

TRAIL is mainly produced by immune cells such as natural killer cells and macrophages, while expression of TRAIL receptors are relatively ubiquitous [4]. In blood vessels, TRAIL receptors are present in both vascular smooth muscle cells (VSMCs) and endothelial cells [5], [6]. In line with these properties, accumulating evidence indicates that TRAIL has a critical role in modulating vascular biology and disease [2]. Indeed, both clinical and animal studies suggest that TRAIL may have a vascular protective role by suppressing the process of atherosclerosis [7]–[10], although the mechanisms of this anti-atherogenic action are not totally understood. In light of these findings, it is proposed that TRAIL not only serves as a biomarker of cardiovascular disease, whereas TRAIL-based therapies may have beneficial pharmacological effects in treating cardiovascular diseases such as atherosclerosis [11].

During atherogenesis, macrophages migrate into the subendothelial space and internalize chemically modified (e.g. oxidized) low-density lipoproteins (LDL), leading to formation of cholesterol-laden foam cells. This process is the central pathophysiological mechanism responsible for the initiation of atherosclerosis [12]. Studies have demonstrated that lipid uptake by macrophages is mediated by various types of scavenger receptors, of which the most functionally important ones include scavenger receptors (SR) class A (SR-AI and -AII ), scavenger receptor-BI (SR-BI), CD36 and lectin-like LDL receptor-1 (LOX-1) [13]–[15]. However, whether TRAIL has any effects on expression of macrophage scavenger receptors and lipid uptake by macrophages has not yet been studied.

Activated macrophage is a main source of TRAIL production, while macrophage functions are also affected by TRAIL. Generally, TRAIL exhibits suppressive effects on normal macrophage functions. For example, using cell culture experiments, people found that TRAIL was capable of inducing macrophage cytotoxicity and trans-differentiation [8], [16], [17]. Moreover, TRAIL receptor signaling is implicated in modulating production of cytokines and activation of NF-κB in stimulated macrophages [18]. Based on these observations, we hypothesized that TRAIL might have anti-atherogenic effects by regulating lipid uptake and scavenger receptor expression in macrophages. To our surprise, however, we found that TRAIL robustly increased the ability of macrophage to internalize modified LDL and the resultant foam cell formation, indicating a possible proatherogenic action.

## Methods

### Ethics Statement

All animal studies were approved by the Qilu Hospital Animal Ethics Committee.

### Reagents

Recombinant human TRAIL was purchased from BioVision (Milpitas California, USA). Recombinant TNF-α was from R&D Systems (Minneapolis, MN, USA). Rabbit anti-SR-AI antibody was from Abcam (Cambridge, UK). Rabbit anti-TRAIL was from Novus Biologicals (Littleton, CO, USA). Rabbit antibodies against ERK, phospho-ERK, p38, phospho-p38, JNK and phospho-JNK were all from Cell Signaling Technology (Beverly, MA, USA). SB202190, JNK Inhibitor II, U0126, BLT-1, staurosporine and z-VAD-fmk were all from Merck Millipore (Darmstadt, Germany). Poly(I:C) was from InvivoGen (San Diego, CA, USA). Lipopolysaccharide (LPS) was from Sigma (St. Louis, MO, USA). DiI-labeled acetylated LDL (DiI-Ac-LDL) and oxidized LDL (ox-LDL) were from Yiyuan Biotechnologies (Guangzhou, China).

### Culture of Cell Lines

Murine macrophage cell line RAW264.7 and human monocytic cell line THP-1, as described previously [19], [20], were obtained from American Type Culture Collection (ATCC) and maintained in DMEM or RPMI 1640 respectively, containing 10% FBS and penicillin/streptomycin (Invitrogen, Carlsbad, CA, USA) in a 5% CO_2_ humidified incubator at 37°C. For THP-1 cell differentiation, cells were seeded in culture plates at 2×10^6^ cells per ml and allowed to adhere and differentiate overnight at 37°C in the presence of 100 nM phorbol myristate acetate (PMA) (Sigma, St. Louis, MO, USA).

### Isolation and Culture of Mouse Primary Peritoneal Macrophages

Wild type C57BL/6 mice were purchased from Vital River Laboratory Animal Technology (Beijing, China). DR5-deficient mice on C57BL/6 background were obtained from Mutant Mouse Regional Resource Centers (MMRRC). Primary peritoneal macrophages were isolated as described [21]. Briefly, 2 ml of 4% sterile Brewer thioglycollate medium (from Sigma) was injected into the peritoneal cavity. After 3 days, elicited macrophages were collected by peritoneal lavage with 5 ml cold PBS and cultured in DMEM supplemented with 10% FBS. After 2 hr incubation to allow for adherence of macrophages, the culture dishes were washed to remove non-adherent cells.

### Isolation and Culture of Mouse Aortic Smooth Muscle Cells

Primary aortic VSMCs were isolated from wild type mice using enzymatic digestion as reported previously, using a mixture containing collagenase I (1 mg ml^−1^), elastase (0.5 mg ml^−1^) and trypsin (1.25 mg ml^−1^) [22]. Smooth muscle cells were cultured in DMEM supplemented with 10% FBS. Cells below passage 8 were used for experimentation.

### Real-time PCR

Total RNA was isolated using Trizol reagent (Invitrogen). cDNA was prepared from 1 µg RNA using TaqMan Reverse Transcription Reagents (Applied Biosystems, Carlsbad, CA, USA) according to the manufacturer’s instructions. Real-time PCR was performed using either Taqman probes (with the TaqMan Gene Expression Master Mix, all from Applied Biosystems), or the SYBR Green method (SsoFas EvaGreen Supermix, from Bio-Rad, Hercules, CA, USA). An ABI Prism 7500 system (Applied Biosystems) was used for PCR amplification and the 2^−ΔΔCT^ method was used to assess the relative mRNA expression level.

### Western Blot Analysis

Total proteins were loaded onto 10% SDS-PAGE gel, separated and electro-blotted onto a nitrocellulose membrane. After blocking with 5% non-fat milk, membranes were probed using various primary antibodies at 4°C for overnight, followed by 2 hr of incubation with 1∶5000 diluted horseradish peroxidase conjugated secondary antibodies at room temperature. The antigen-antibody complexes were detected by ECL Prime western blotting detection reagent from GE Healthcare (Pittsburgh, PA, USA).

### Analysis of Lipid Uptake and Foam Cell Formation

RAW264.7 or differentiated THP-1 cells were cultured on Lab-Tek II chamber slides (Thermo Scientific, Pittsburgh, PA, USA) and loaded with DiI-Ac-LDL (30 µg ml^−1^) for 2, 4 or 8 hr. Then cells were fixed with 4% paraformaldehyde at room temperature for 20 min. After 3 rinses, cells were counterstained with DAPI. Fluorescent images were obtained with a confocal microscope (Model LSM710, Zeiss, Jena, Germany) at 549 nm excitation and 565 nm emission. To assess macrophage transformation into foam cells, we analyzed the accumulation of intracellular lipid droplets using Oil Red O staining. Briefly, cells cultured on slides were loaded with ox-LDL (80 µg ml^−1^) for 48 hr, then cells were fixed with 4% paraformaldehyde, and stained with Oil Red O (0.3% dissolved in isopropyl alcohol) for 15 min at room temperature. Hematoxylin was used for counterstaining. Slides were evaluated by light microscopy.

### Analysis of Apoptosis

Terminal deoxynucleotidyl transferase-mediated dUTP nick end labeling (TUNEL) was performed for detection and quantification of apoptosis, using ApopTag Plus Peroxidase In Situ Apoptosis Detection Kit (Merck Millipore) following the manufacturer’s protocol. The number of TUNEL-positive cells were counted and averaged across 10 random fields from 3 independent experiments each.

### RNA Interference

Synthetic siRNA for mouse SR-AI (5′-GGAGGAACGUGUGUACAAATT-3′) was purchased from GenePharma (Shanghai, China). A non-targeting siRNA (5′-UUCUCCGAACGUGUCACGUTT-3′) was used as control. For transfection, adherent cells of 60–70% confluent were maintained in antibiotic-free Opti-MEM medium and incubated with a mixture of siRNA and Lipofectamine RNAiMAX reagent (5 µl ml^−1^) (Invitrogen). The final concentration of siRNA was 200 nM. After 6 hr of treatment, the cells were changed to fresh complete culture medium, and incubated for additional 48 hr.

### Statistical Analysis

All experiments were repeated at least three times. Data are presented as mean ± standard error of the mean (SEM). Data analysis was performed with unpaired *t*-test or one-way ANOVA followed by *post hoc* Newman-Keuls test. *P*<0.05 was considered as statistically significant.

## Results

### TRAIL Promotes Lipid Uptake and Foam Cell Formation in Macrophages

**Figure 1:**
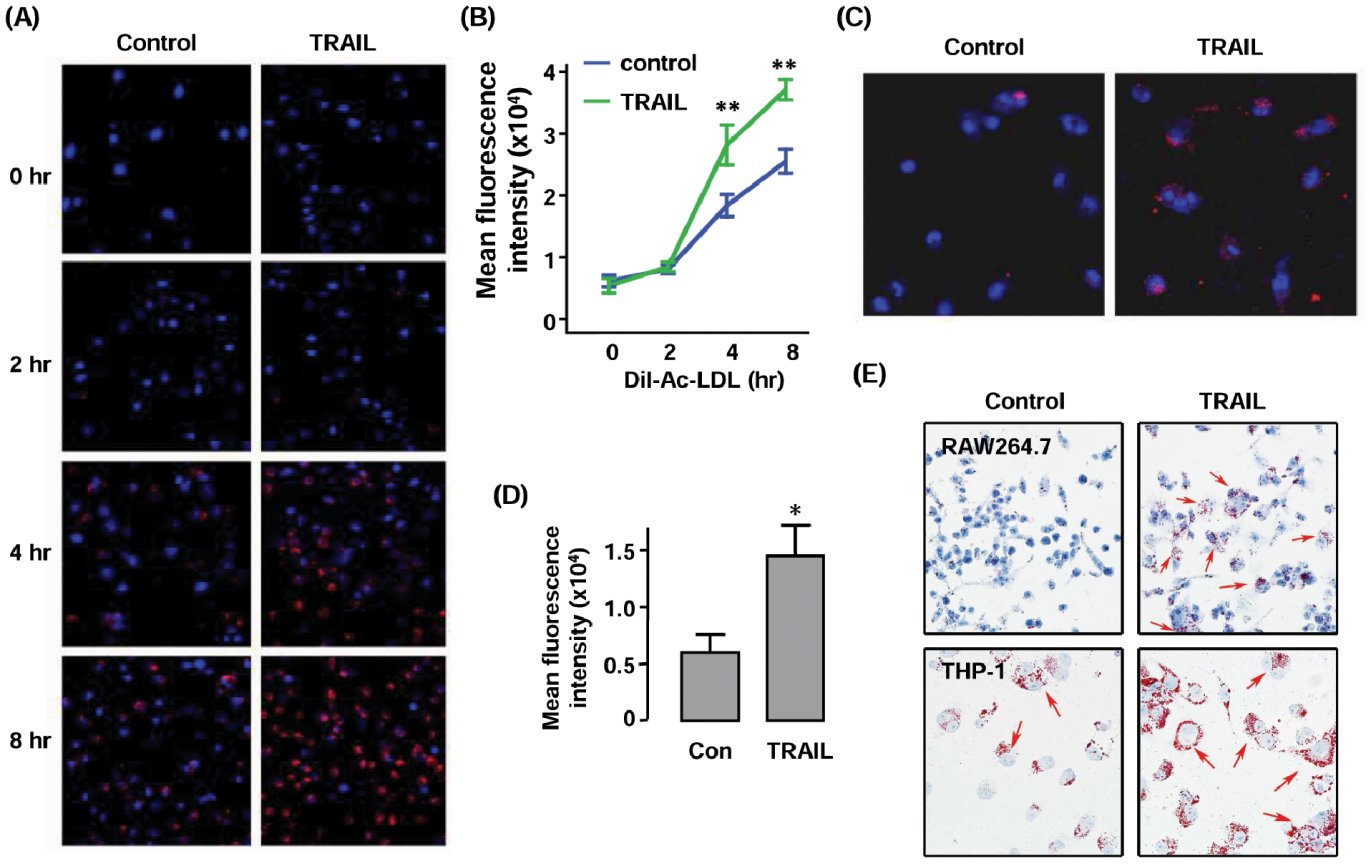
Effects of TRAIL on lipid uptake and foam cell formation in macrophages. (**A**) Fluorescence images showing the effects of TRAIL on DiI-Ac-LDL (red) uptake in RAW264.7 cells. Cells were pretreated with TRAIL (10 ng ml^−1^) for 24 hr, then loaded with DiI-Ac-LDL for different time as indicated. (**B**) Quantitative data of the fluorescence intensity in (A). (**C & D**) Effects of TRAIL (10 ng ml^−1^ for 24 hr) on DiI-Ac-LDL uptake in PMA-differentiated human THP-1 cells. (**E**) Effects of TRAIL on ox-LDL-triggered foam cell formation in RAW264.7 and THP-1 cells as detected by Oil Red O staining (red color, arrows). * *P*<0.05 and ** *P*<0.01 *vs* control, unpaired *t*-test or one-way ANOVA followed by Newman-Keuls test, *n* = 3.

We first evaluated the effects of TRAIL on DiI-Ac-LDL uptake in RAW264.7 cells. We found that pretreatment with TRAIL (10 ng ml^−1^) for 24 hr significantly increased intracellular DiI-Ac-LDL accumulation as measured by fluorescent microscopy (Figure 1A & 1B). To clarify whether this stimulatory effect on lipid uptake was also present in human cells, we repeated the experiment in differentiated human THP-1 cells. As shown in Figure 1C & 1D, TRAIL exhibited a similar increasing effect on DiI-Ac-LDL accumulation in human macrophages. To further confirm that the effects of TRAIL on macrophage lipid uptake were relevant to foam cell formation, we pretreated RAW264.7 and THP-1 cells with TRAIL and then loaded the cells with ox-LDL for a longer period of 48 hr. Oil Red O staining clearly demonstrated that TRAIL treatment increased the amount of intracellular lipid droplets (Figure 1E). These results demonstrated that TRAIL induced robust stimulatory effects on lipid uptake and foam cell formation in cultured macrophages.

### The Effect of TRAIL on Foam Cell Formation was Absent in DR5-deficient Macrophages

**Figure 2:**
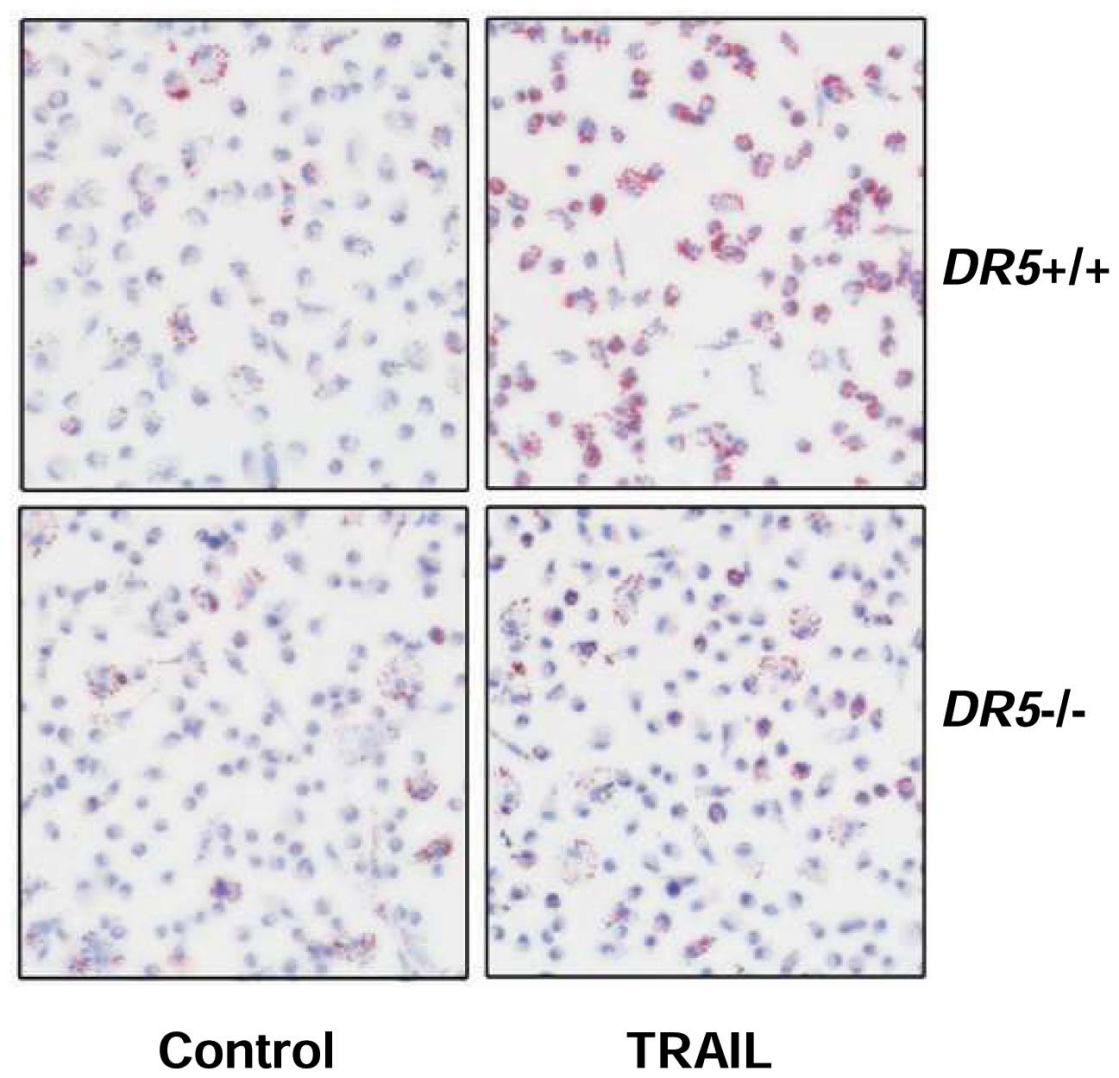
Effects of TRAIL on foam cell formation in wild type (DR5+/+) and DR5-deficient (DR5−/−) peritoneal macrophages. Cells were treated with TRAIL at 10^−1^ for 24 hr, and then loaded with ox-LDL (80 µg ml^−1^) for 48 hr. Internalized LDL was detected with Oil Red O staining (red color).

Using recombinant TRAIL could be complicated by contamination of bacterial endotoxins. To exclude the possibility that the observed effects of TRAIL were caused by endotoxin contaminations, and to further clarify the specific role of TRAIL receptors in mediating the observed effects, we isolated wild type and DR5-deficient peritoneal macrophages (Figure S1) and measured ox-LDL-induced foam cell formation with and without TRAIL pretreatment. We showed that similar to cell lines, primary macrophages of wild type exhibited increased foam cell formation in response to TRAIL treatment, whereas this effect of TRAIL was totally abolished in DR5-deficient macrophages (Figure 2).

### TRAIL Upregulated the Expression of SR-AI and SR-BI in Macrophages

**Figure 3:**
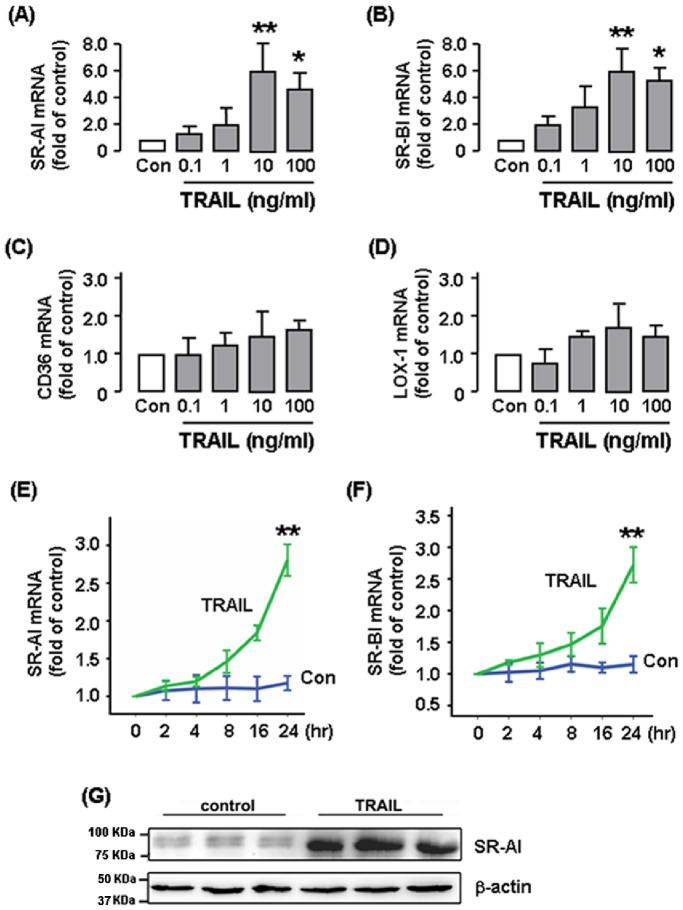
Effects of varying concentrations of TRAIL on expressions of different scavenger receptors in RAW264.7 cells. Cells were treated with TRAIL for 24(**A to D**) Quantitative real-time PCR results of mRNA expression of scavenger receptors. (**E & F**) Time course of SR-AI and SR-BI expression in cells treated with TRAIL (10 ng ml^−1^). (**G**) Western blot showing the effect of TRAIL on SR-AI protein expression (*n* = 3 independent experiments). The PCR results are expressed as fold of control (Con). *P<0.05 *vs* Con, **P<0.01 *vs* Con, one-way ANOVA, *n* = 3–6.

**Figure 4:**
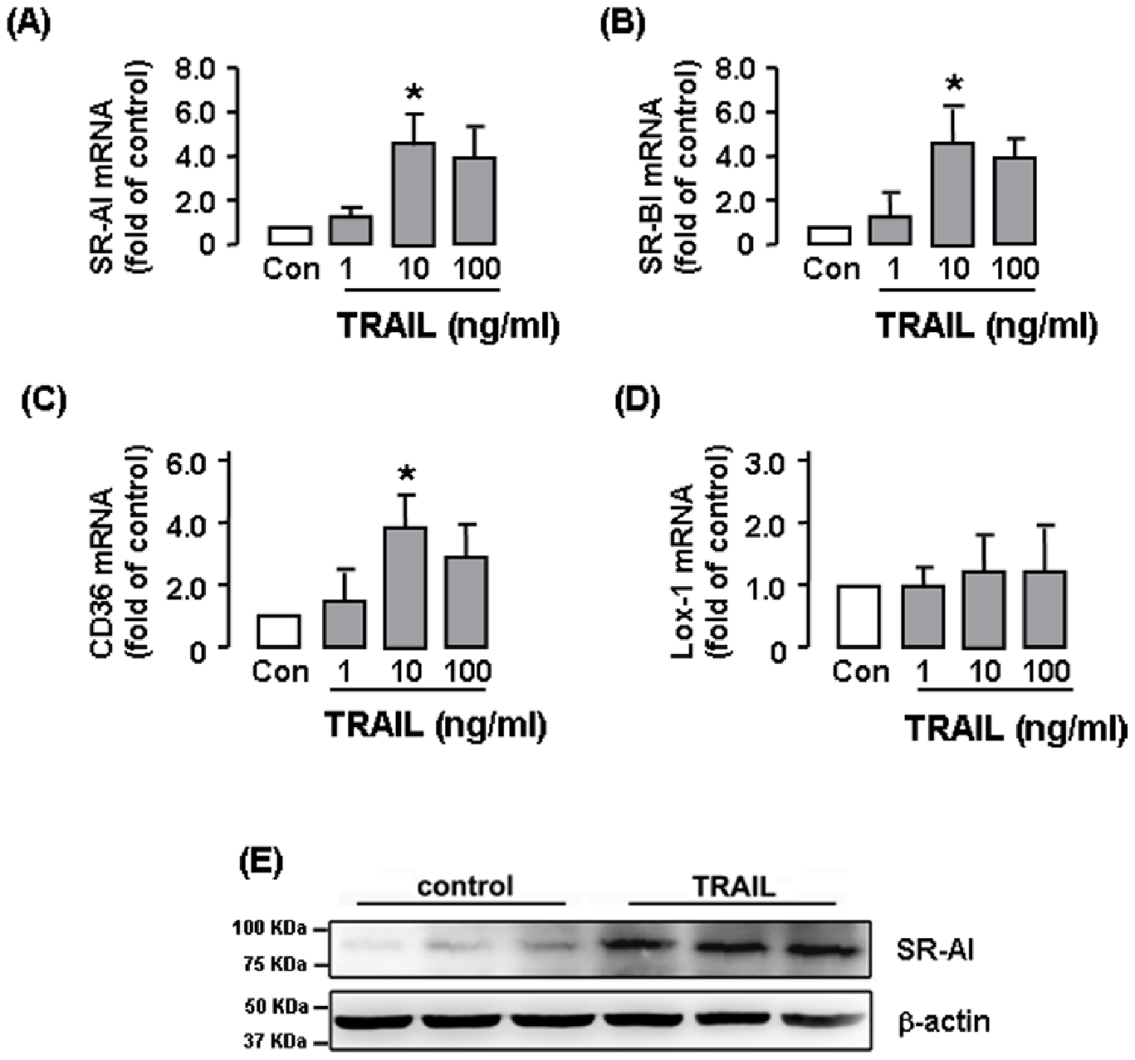
Effects of varying concentrations of TRAIL on expressions of scavenger receptors in THP-1 cells. (**A to D**) Quantitative real-time PCR results of mRNA expression of scavenger receptors. (**E**) Western blot showing the effect of TRAIL on SR-AI protein expression (*n* = 3 independent experiments). The PCR results are expressed as fold of control (Con). * *P*<0.05 *vs* Con, one-way ANOVA, *n* = 3–6.

We next evaluated the effects of TRAIL on expression of various scavenger receptors using real-time PCR. In RAW264.7 cells, incubation with TRAIL from 0.1–100 ng ml^−1^ for 24 hr significantly increased expressions of SR-AI and SR-BI in a concentration-dependent manner (Figure 3A and 3B). The responses were maximum at 10 ng ml^−1^, while further increasing the concentration to 100 ng ml^−1^ slightly weakened the effects. In contrast to SR-AI and SR-BI, TRAIL had no significant effects on expressions of CD36 or LOX-1 (Figure 3C and 3D). To examine the time course of TRAIL-stimulated scavenger receptor expression, we treated the cells with TRAIL at 10 ng ml^−1^ for various time, and demonstrated that TRAIL induced a gradual response over time with the effects being most significant at 24 hr (Figure 3E and 3F). However, no further increase in the response was observed beyond 24 hr (data not shown). We also performed western blot to measure the SR-AI protein expression. As shown in Figure 3G, TRAIL (10 ng ml^−1^ for 24 hr) markedly increased the SR-AI protein expression. To confirm the effects of TRAIL in human cells, we repeated the experiments in THP-1 cells. As demonstrated in Figure 4, TRAIL produced very similar responses in scavenger receptor expression as in murine cells, apart from that TRAIL also induced upregulation of CD36 in THP-1 cells.

**Figure 5:**
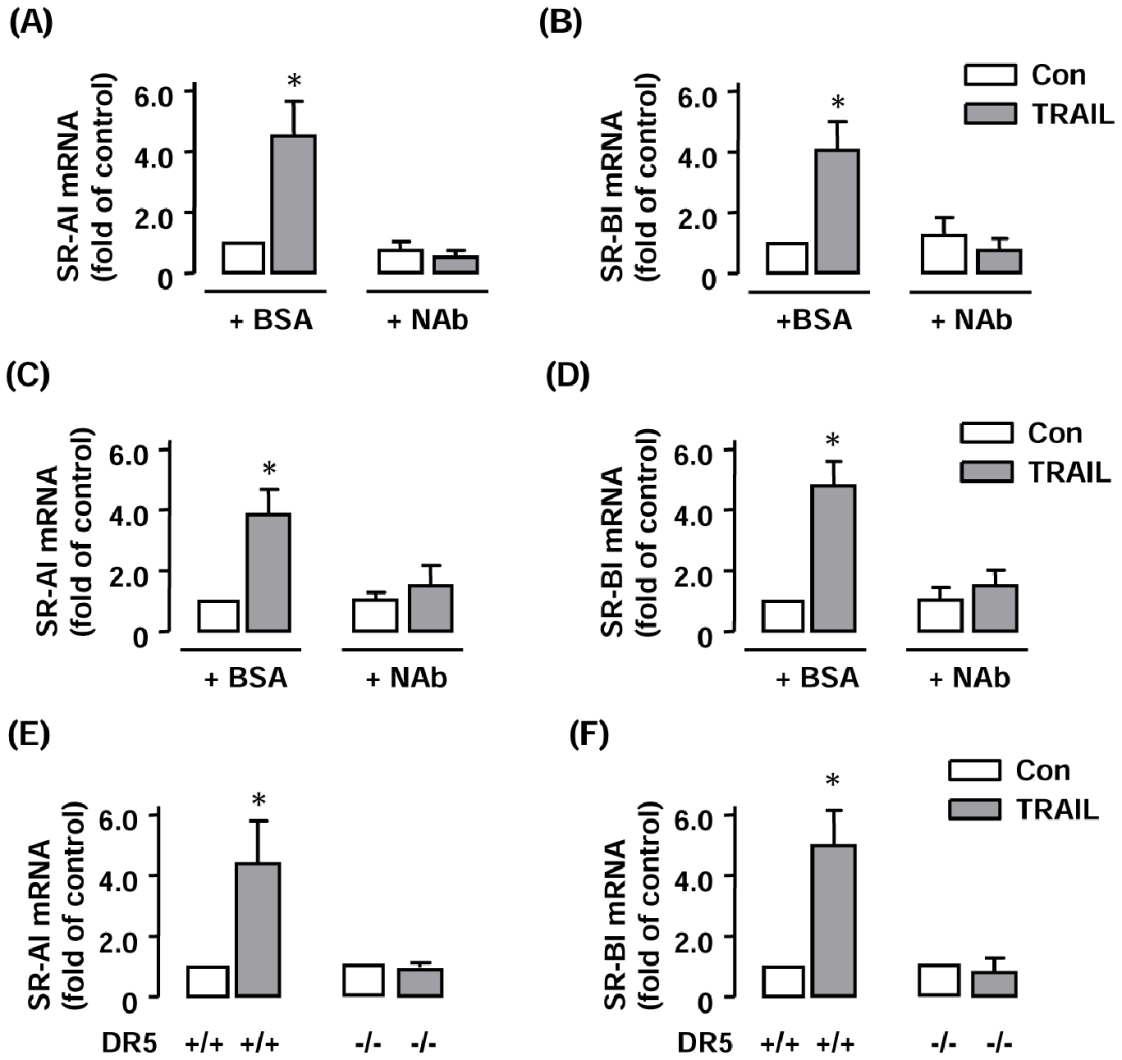
TRAIL-stimulated scavenger receptor expressions were blocked by TRAIL neutralizing antibody or DR5 deficiency. (**A to D**) Blockade of the effects of TRAIL on SR-AI and SR-BI expression with a TRAIL neutralizing antibody (NAb) (5 µg ml^−1^) in RAW264.7 (A & B) and THP-1 cells (C & D). Bovine serum albumin (BSA) in saline buffer was used as vehicle control. (**E & F**) The effects of TRAIL on SR-AI and SR-BI expression were abolished in DR5-deficient peritoneal macrophages. * *P*<0.05 *vs* control (Con), one-way ANOVA or unpaired *t*-test, *n* = 3–4.

To confirm the specificity of TRAIL effects on scavenger receptor expression, we performed blocking experiments by co-incubating the cells with a polyclonal antibody against TRAIL. As shown in Figure 5A-5D, the TRAIL antibody (at 5 µg ml^−1^) completely abolished the stimulatory effects of TRAIL on SR-AI and SR-BI expressions in both RAW264.7 and THP-1 cells. In addition, we demonstrated that TRAIL-induced effects on SR-AI and SR-BI expression were totally absent in DR5-deficient macrophages as compared to wild type cells (Figure 5E and 5F). Moreover, we compared the effects of TRAIL on SR-AI and SR-BI expression with those induced by TNF-α in THP-1 cells. Interestingly, we found that treating cells with TNF-α (20 ng ml^−1^) for 24 hr significantly decreased the expression of SR-AI but increased the expression of SR-BI in THP-1 cells (Supplemental Figure S2).

### TRAIL-stimulated Lipid Uptake was Mediated by SR-A

**Figure 6:**
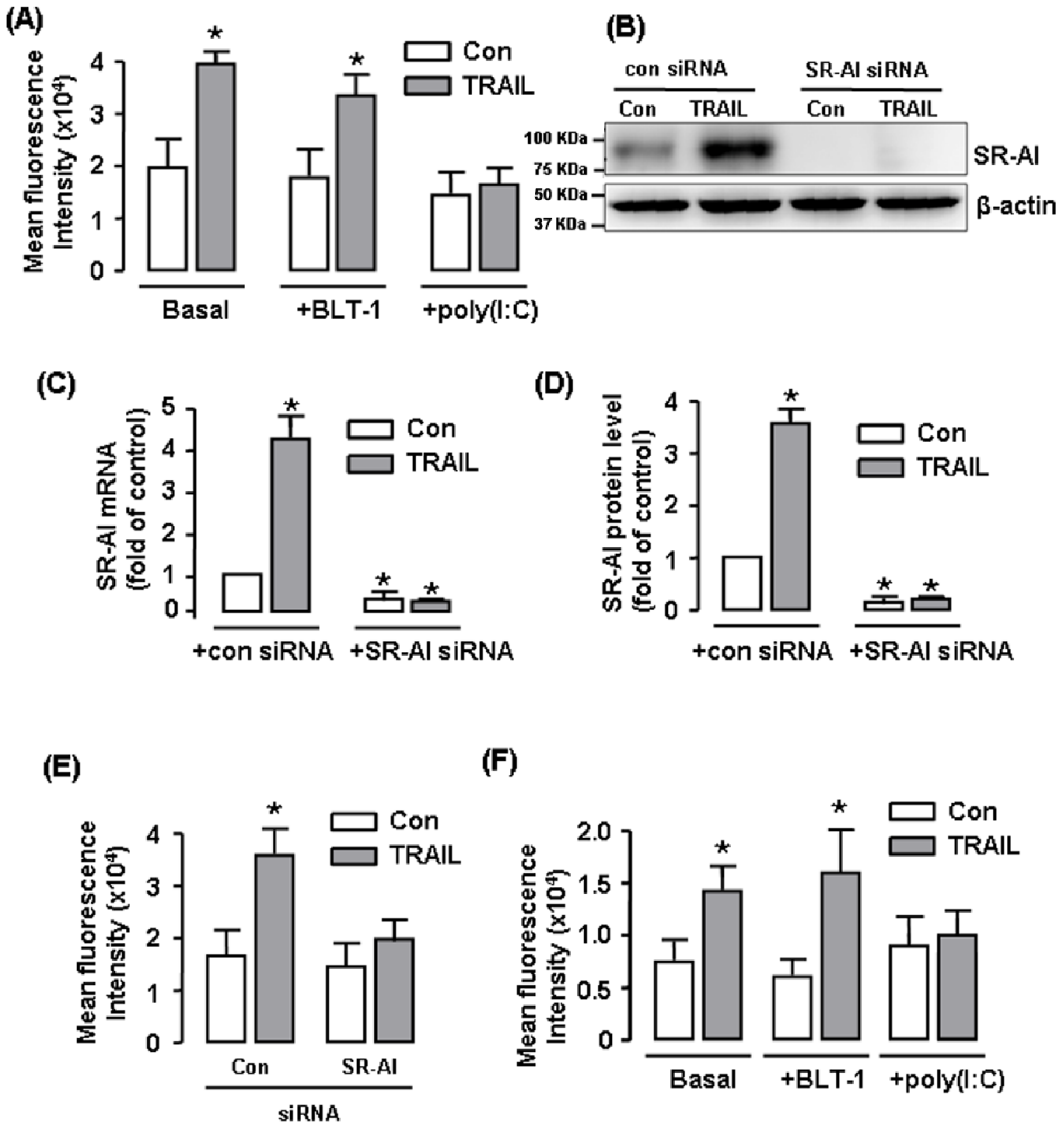
Effects of blockade of SR-AI and SR-BI functions on TRAIL-stimulated DiI-Ac-LDL uptake in macrophages. (**A**) Effects of the SR-AI inhibitor poly(I:C) (1 µM) and the SR-BI inhibitor BLT-1 (5 µM) on TRAIL-stimulated DiI-Ac-LDL uptake in RAW264.7 cells. (**B**) Western blot showing the effect of siRNA on protein expression of SR-AI in RAW264.7 cells. Cells were incubated with control or SR-AI siRNA (all at 200 nM) for 48 hr. (**C & D**) Quantitative data showing the knocking down effects of siRNA on SR-AI mRNA and protein levels measured by real-time PCR or western blot (**E**) SR-AI siRNA blocked the stimulating effect of TRAIL on DiI-Ac-LDL uptake in RAW264.7 cells. (**F**) Effects of poly(I:C) and BLT-1 on TRAIL-stimulated DiI-Ac-LDL uptake in THP-1 cells. * *P*<0.05 *vs* control (Con), one-way ANOVA or unpaired *t*-test, *n* = 3–4.

To test the roles of SR-AI and SR-BI in TRAIL-induced foam cell formation, we examined the effects of the SR-A inhibitor poly(I:C) (1 µM) and the SR-BI inhibitor BLT-1 (5 µM) on TRAIL-stimulated DiI-Ac-LDL uptake in RAW264.7 cells. We found that pretreatment with poly(I:C), but not BLT-1, significantly blunted the augmenting effects of TRAIL on DiI-Ac-LDL uptake (Figure 6A and Figure S3), suggesting that SR-A was involved in mediating the TRAIL effects. To further confirm the specific role of SR-AI, we transfected RAW264.7 cells with SR-AI siRNA. We screened three different siRNA sequences and selected the one with highest efficacy of reducing both of the mRNA and protein levels of SR-AI (Figure 6B-6D). Similar to the effects of poly(I:C), SR-AI siRNA significantly suppressed the stimulating effect of TRAIL on DiI-Ac-LDL accumulation (Figure 6E). The specific role of SR-AI in mediating TRAIL-induced lipid uptake was also confirmed in human THP-1 cells (Figure 6F and Figure S3).

### TRAIL-stimulated SR-AI Expression Requires p38 Activation

**Figure 7:**
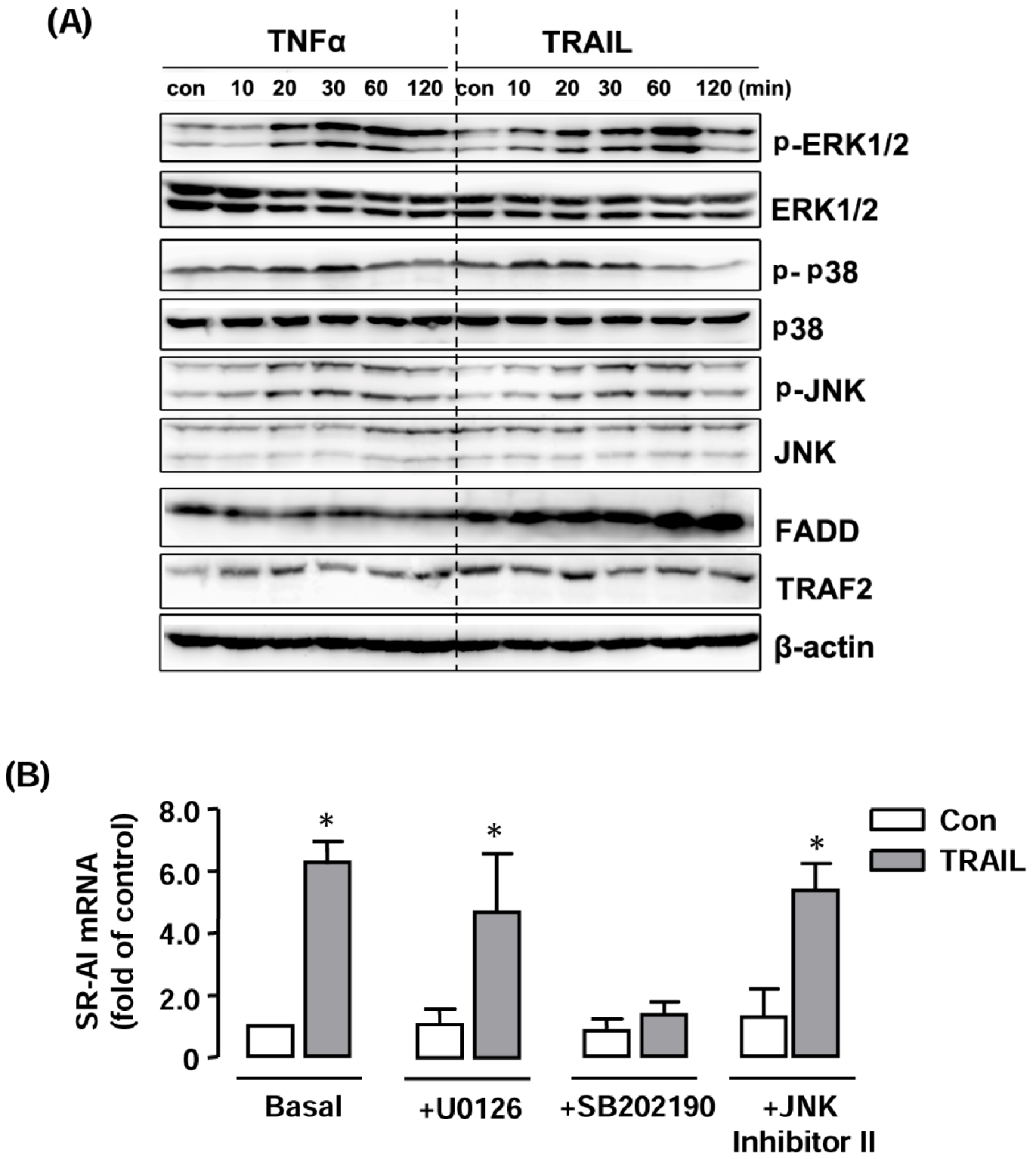
Role of MAPKs in TRAIL-induced SR-AI expression. (**A**) Western blot showing the effects of TRAIL (10 ng ml^−1^) on phosphorylation of ERK1/2, p38 and JNK MAPKs in RAW264.7 cells. TNF-α (20 ng ml^−1^) was used as a positive control. The total levels of FADD or TRAF2 were not changed. (**B**) Effects of the ERK pathway inhibitor U0126 (1 µM), p38 inhibitor SB202190 (1 µM) and JNK Inhibitor II (1 µM) on TRAIL-stimulated SR-AI expression. The results are expressed as fold of control (Con). * *P*<0.05 *vs* Con, one-way ANOVA, *n* = 3.

To clarify the mechanisms of TRAIL-induced effects on SR-AI gene expression, we first examined the effects of TRAIL on activation of MAPKs in RAW264.7 cells. As shown in Figure 7A, treatment with TRAIL triggered phosphorylation of ERK1/2, p38 and JNK. These effects of TRAIL on MAPK signaling were similar to those induced by TNF-α, which was used as a positive control (Figure 7A). The total levels of FADD or TRAF2 were not changed. Pretreatment of the cells with the selective p38 inhibitor SB202190, but not U0126 (ERK pathway inhibitor) or JNK Inhibitor II, blunted TRAIL-induced upregulation of SR-AI expression (Figure 7B).

### Effects of TRAIL on Macrophage Apoptosis

**Figure 8:**
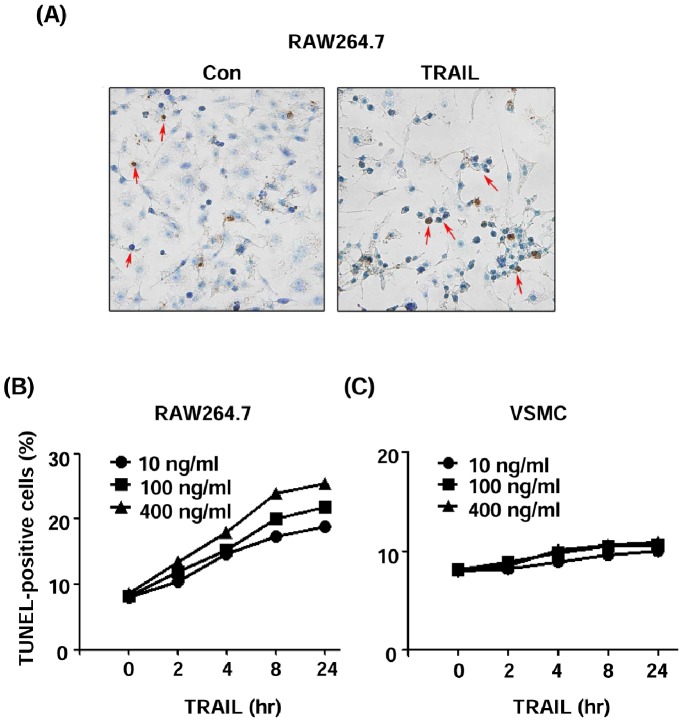
Effects of TRAIL on apoptosis of macrophage and vascular smooth muscle cells (VSMCs). (**A**) Representative images of TUNEL labeling showing apoptotic cells (arrows) in control and TRAIL-treated (100 ng ml^−1^ for 24 hr) RAW264.7 cells. (**B**) Quantitative analysis of TUNEL-positive RAW264.7 cells after treatment with TRAIL (10, 100 and 400 ng ml^−1^) for various time as indicated. (**C**) Quantitative analysis of TUNEL-positive cells in VSMCs after treatment with TRAIL for different time. Data are % of total cells averaged from triplicate experiments.

We also tested the effects of TRAIL on apoptosis of macrophages. As shown in Figure 8A and 8B, TRAIL of concentrations from 10–400 ng ml^−1^ all effectively induced apoptosis in a time-dependent manner in RAW264.7 cells as assessed by TUNEL assay.

### Effects of TRAIL on Scavenger Receptor Expression and Apoptosis in VSMCs

To test whether the observed effects of TRAIL on scavenger receptor expression and apoptosis were specific to macrophages, we measured the mRNA expression of SR-AI, SR-BI, LOX-1 or CD36 in mouse cultured VSMCs. In contrast to macrophages, VSMCs did not express detectable level of any of these receptors. Treatment with TRAIL (10 ng ml^−1^ for 24 hr) did not induce expression of these receptors in VSMCs (data not shown). Moreover, TRAIL had minor effects on apoptosis of VSMCs as compared to macrophages (Figure 8C).

### TRAIL-induced Lipid Uptake was not Dependent on Apoptosis

**Figure 9:**
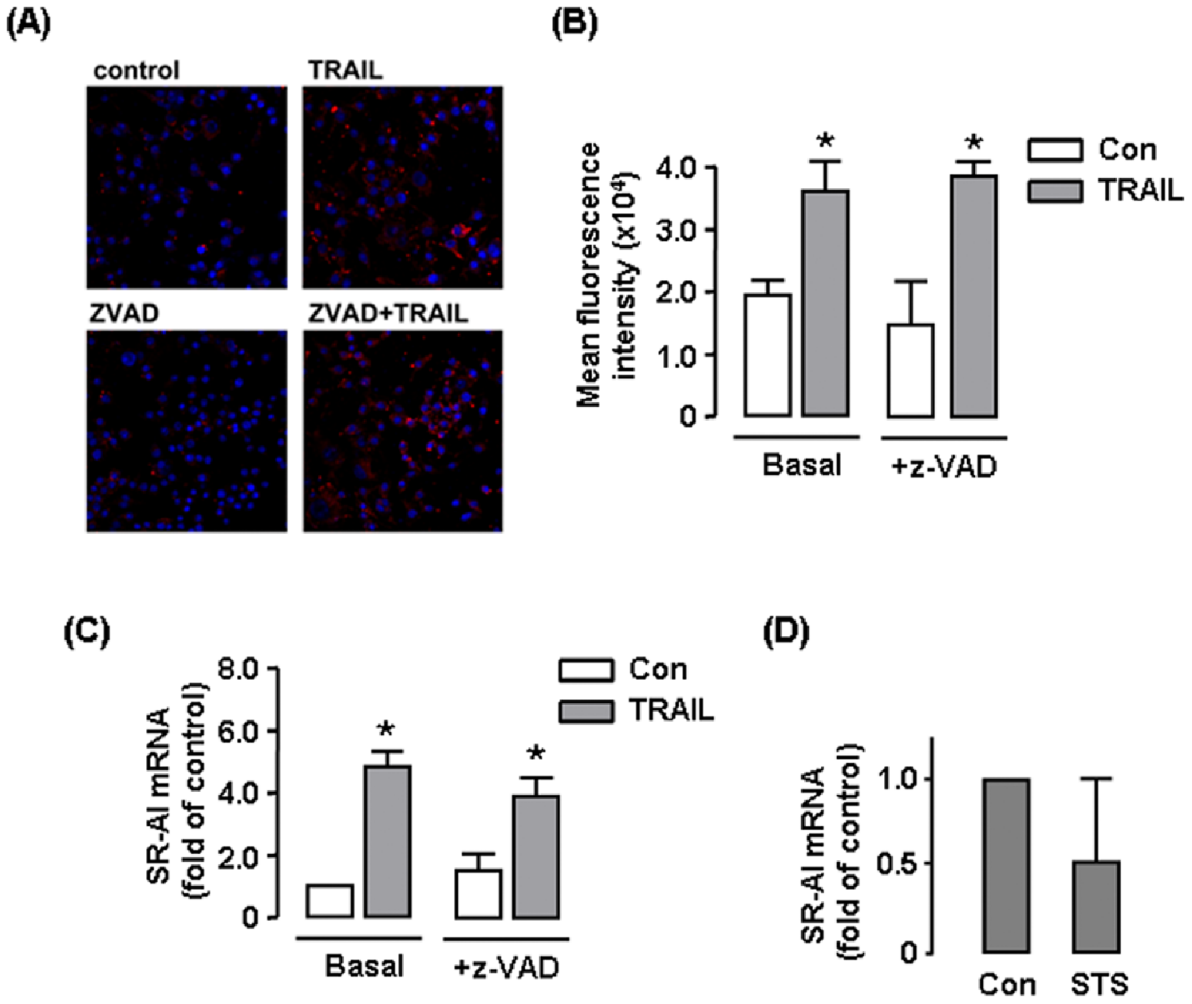
TRAIL-stimulated lipid uptake and SR-AI expression were not dependent on cell apoptosis. (**A**) Effect of co-treatment with the pan-caspase inhibitor z-VAD-fmk (1 µM) on TRAIL (10 ng ml^−1^)-stimulated DiI-Ac-LDL uptake in RAW264.7 cells. (**B**) Quantitative data of the fluorescence intensity in A. (**C**) Effect of z-VAD-fmk on TRAIL-stimulated SR-AI mRNA expression. (**D**) Effect of staurosporine (STS, 1 µM)-induced apoptosis on SR-AI expression. Cells were treated with STS for 1 hr and then incubated in fresh medium for additional 24 hr. * *P*<0.05 *vs* control (Con), one-way ANOVA, *n* = 3.

Because TRAIL induced apoptotic responses in macrophages, next we tested whether the effects of TRAIL on macrophage lipid uptake was dependent on cell apoptosis. First we pretreated the cells with the pan-caspase inhibitor z-VAD-fmk (1 µM) [23], which diminished the pro-apoptotic effect of TRAIL in RAW264.7 cells. We found that z-VAD-fmk had no significant effects on the basal level or TRAIL-stimulated lipid accumulation or SR-AI expression (Figure 9A–9C). Then we treated the cells with staurosporine (1 µM) for 1 hr [24], which induced ∼ 20–30% cell apoptosis, a response similar to that induced by TRAIL. Staurosporine was washed out and cells incubated for additional 24 hr. We found that staurosporine tended to decrease the expression of SR-AI (*P*>0.05) (Figure 9D). Staurosporine did not show any increasing effect on lipid accumulation in RAW264.7 cells (data not shown). Taken together, these observations suggested that TRAIL-induced foam cell formation was independent of the presence of cell apoptosis.

## Discussion

In the present study, we provided evidence that the cytokine TRAIL has stimulatory effects on macrophage lipoprotein internalization and foam cell formation in vitro, which were observed in both mouse and human macrophage cell lines and mouse primary macrophages. To our knowledge, this is the first characterization of the pharmacological effects of TRAIL on lipid uptake functions in macrophages. Moreover, we showed that TRAIL significantly increased the expression of SR-AI and SR-BI. In addition, only inhibition of the function of SR-A, but not that of SR-BI, blocked TRAIL-stimulated lipid uptake, suggesting that the TRAIL effects on macrophage foam cell formation were mediated by upregulation of SR-AI expression. While the expression of LOX-1 was not altered by TRAIL, it significantly increased expression of CD36 in human macrophages but not in murine cells. We also showed that all of the effects of TRAIL on SR-AI expression and lipid internalization were mediated by the cognate receptor DR5, as TRAIL showed no effects in DR5-deficient macrophages. The results with DR5−/− cells can also rule out the possibility that endotoxin contamination contributes to the observed effects induced by the recombinant TRAIL peptide. We further confirmed this using a specific antibody to TRAIL, which blocked the effects of TRAIL on SR-AI expression.

Experimental evidence has suggested that SR-A receptors are responsible for 80% of Ac-LDL uptake in macrophages [25]. This is consistent with our results that TRAIL-stimulated Ac-LDL internalization is largely blocked by SR-AI but not SR-BI inhibition. Despite that initial studies have shown that SR-AI is highly expressed in macrophage-rich areas of human atherosclerotic lesions, and SR-A knockout mouse exhibits a decrease in atherogenesis, whether SR-A upregulation can be directly linked to enhanced atherogenesis in vivo is still uncertain [15], [25]. This may be related to that, apart from mediating lipoprotein uptake by macrophages, SR-A also has other important biological functions that are independent of lipid transportation [25]. On the other hand, there is evidence suggesting that CD36 plays a major role in macrophage ox-LDL internalization, contributing 60–70% of cholesterol ester accumulation in macrophages exposed to ox-LDL [26]. We found that TRAIL significantly upregulated CD36 expression in human THP-1 cells, raising the possibility that CD36 might also be involved in foam cell formation in human macrophages stimulated with TRAIL. The near total blockade of Ac-LDL uptake in human cells by poly(I:C) may be explained by the relatively low affinity of CD36 to Ac-LDL as compared to SR-AI [13]–[15]. Nonetheless, like SR-AI, the precise role of CD36 in the development of atherosclerosis in vivo is still inconclusive [15].

Previous studies using TRAIL-deficient mice demonstrated that absence of TRAIL aggravated the process of atherosclerotic lesion formation on apolipoprotein E-deficient background, indicating that endogenous TRAIL may have a protective role against atherogenesis [9], [10]. However, it is also noted that in patients with inflammatory diseases such as psoriatic arthritis, systemic lupus erythematosus, and viral infection, the circulating level of TRAIL is elevated [27]–[29]. It is recognized that a chronic systemic inflammatory condition caused by these diseases may increase the risk of atherosclerosis [30]. Moreover, several lines of in vitro experiments demonstrated that exogenous TRAIL triggered prominent inflammatory reactions in both vascular endothelial and smooth muscle cells [31], [32]. Taken these results together, our data argue that the precise role of TRAIL during atherogenesis, especially at concentrations in excess to the normal physiological level, remains to be determined.

TRAIL and TNF-α utilize similar intracellular signaling pathways to modulate cell functions. In particular, NF-κB and MAPK pathways are the major down stream effectors of TRAIL and TNF-α signaling [1], [2], [4]. However, previous studies showed that TNF-α treatment decreased the expression SR-A in THP-1 cells, whereas it showed no significant effect in RAW264.7 cells [33], [34]. In the present study, we confirmed that TRAIL and TNF-α indeed had divergent effects on SR-AI expression in THP-1 cells. However, the mechanisms of this discrepancy are not clear. We found that the effect of TRAIL on SR-AI expression showed a bell-shaped concentration-response relationship, with the maximal effect being observed at 10 ng ml^−1^, while further increasing its concentration produced less effects. Interestingly, a narrow concentration-response relationship is also observed in TNF-α-induced responses; and in some circumstances, different concentrations of TNF-α may result in opposite cellular outcomes [35]–[38]. Nevertheless, to our knowledge, a full concentration-response analysis has not been carried out yet in characterization of the modulating effects of TNF-α on macrophage scavenger receptor expression. The signaling mechanisms by which TNF-α suppresses SR-A expression is unclear, and this may involve post transcriptional regulations [33]. Of note, the components of the signaling complexes formed following TRAIL or TNF-α ligation to their cognate receptors are not entirely identical. Moreover, the dynamics of signal initiation and termination at the receptor level are also distinct between TRAIL and TNF-α [4]. Based on these data, it is possible that in addition to the activation of pathways involved in stimulating SR-A gene transcription as TRAIL does, TNF-α may concomitantly activate other pathways that promote SR-A mRNA degradation [33]; a potential stimulating action of TNF-α on SR-A transcription could be effectively masked by the latter effect. This hypothesis is supported by an observation that TNF-α induced significant SR-A mRNA upregulation in human arterial endothelial cells, indicating that TNF receptor-mediated signaling is indeed capable of stimulating SR-A transcription [39]. Interestingly, a recent study demonstrated that NF-like protein 1A, a TNF super family cytokine with similar intracellular signaling mechanisms as TNF-α and TRAIL [40], [41], also promoted foam cell formation in human macrophages [42].

Transcription of the SR-AI gene in macrophages is regulated by a variety of signaling pathways. There is evidence that macrophage-specific expression of SR-AI is controlled by the ETS family transcription factor PU.1/Spi-1 [43]. In addition, multiple AP-1 binding sites have been identified in the promoter region of SR-AI gene, while AP-1 and ETS2 have been shown to have synergistic actions in driving SR-AI transcription in macrophages [44]. In line with these findings, Mietus-Snyder *et al.* reported that in smooth muscle cells, c-Jun/JNK might be involved in stimulating SR-A gene expression in response to oxidative stress [45]. In contrast, our study in macrophages showed that TRAIL-induced SR-AI expression was sensitive to inhibition of p38 MAPK, but not JNK or ERK1/2. Indeed, this observation is consistent with several lines of studies showing that p38, but not JNK or ERK1/2, has a critical role in mediating the upregulation of macrophage SR-AI expression induced by various agonists [46]–[49]. However, the molecular mechanisms by which p38 modulates SR-AI expression remain to be elucidated.

TRAIL-mediated apoptosis of macrophage cells has been observed both in vitro and in vivo [8], [50]. In this study, we demonstrated that TRAIL at concentrations that promote foam cell formation can also induce an apoptotic response in macrophages. This proapoptotic effect in macrophages is in contrast to that in VSMCs, in which TRAIL shows a minor effect in induction of apoptosis. This finding is consistent with previous reports that the apoptosis-inducing activity of TRAIL in VSMCs is relatively weak [51]. However, the contribution of TRAIL in VSMC apoptosis *in vivo*, especially in the atherosclerotic plaques, appears to be controversial [8], [52]. Some evidence has suggested that TRAIL-mediated macrophage apoptosis may result in a reduction of the mass of atherosclerotic plaques [8], however, it is also noted that excessive macrophage apoptosis in advanced lesions may result in plaque destabilization [53].

In summary, we have provided evidence showing that TRAIL promotes lipid uptake and foam cell formation in cultured macrophage cells, and this effect is mediated by SR-AI upregulation through activation of the p38 MAPK pathway.

## Supporting Information

Figure S1 **DNA gel image showing the genotyping results of wild type, heterozygous and homozygous DR5-deficient animals.**
(PPT)

Figure S2 Effects of TNF-α (20 ng/ml) on SR-AI and SR-BI expression in THP-1 cells. The results are expressed as fold of control (Con). * ***P*** **<0.05** ***vs*** **Con, unpaired** ***t*** **-test,** ***n*** **= 3.**
(PPT)

Figure S3 **Effects of poly(I:C) and BLT-1 on rTRAIL-stimulated DiI-Ac-LDL uptake.** Fluorescence microscopy images showing the effects of the SR-AI inhibitor poly(I:C) (1 µM) and the SR-BI inhibitor BLT-1 (5 µM) on rTRAIL-stimulated DiI-Ac-LDL uptake in RAW264.7 cells **(A)** and THP-1 cells **(B)**.
(PPT)
