# Supplementary figures and images for: TRAIL/DR5 Signaling Promotes Macrophage Foam Cell Formation by Modulating Scavenger Receptor Expression
Source: PLoS One. 2014 Jan 22;9(1):e87059. doi: 10.1371/journal.pone.0087059 (PMC3899365; doi:10.1371/journal.pone.0087059)

## Slide 1
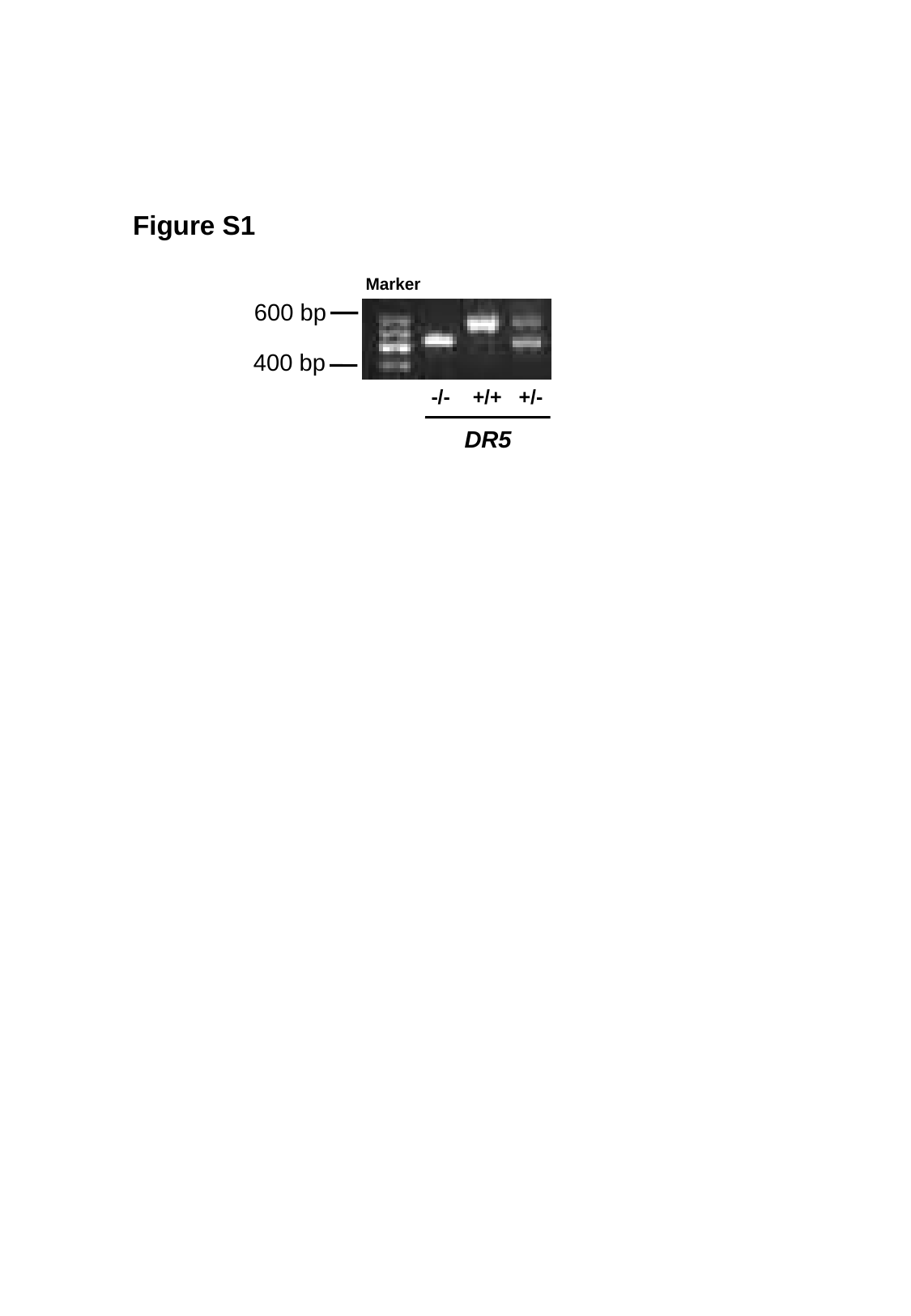

Figure S1
Marker
600 bp
400 bp
 -/-
+/+
+/-
DR5

Supplement: Figure S1 — DNA gel image showing the genotyping results of wild type, heterozygous and homozygous DR5-deficient animals. (PPT) [file pone.0087059.s001.ppt]

## Slide 1
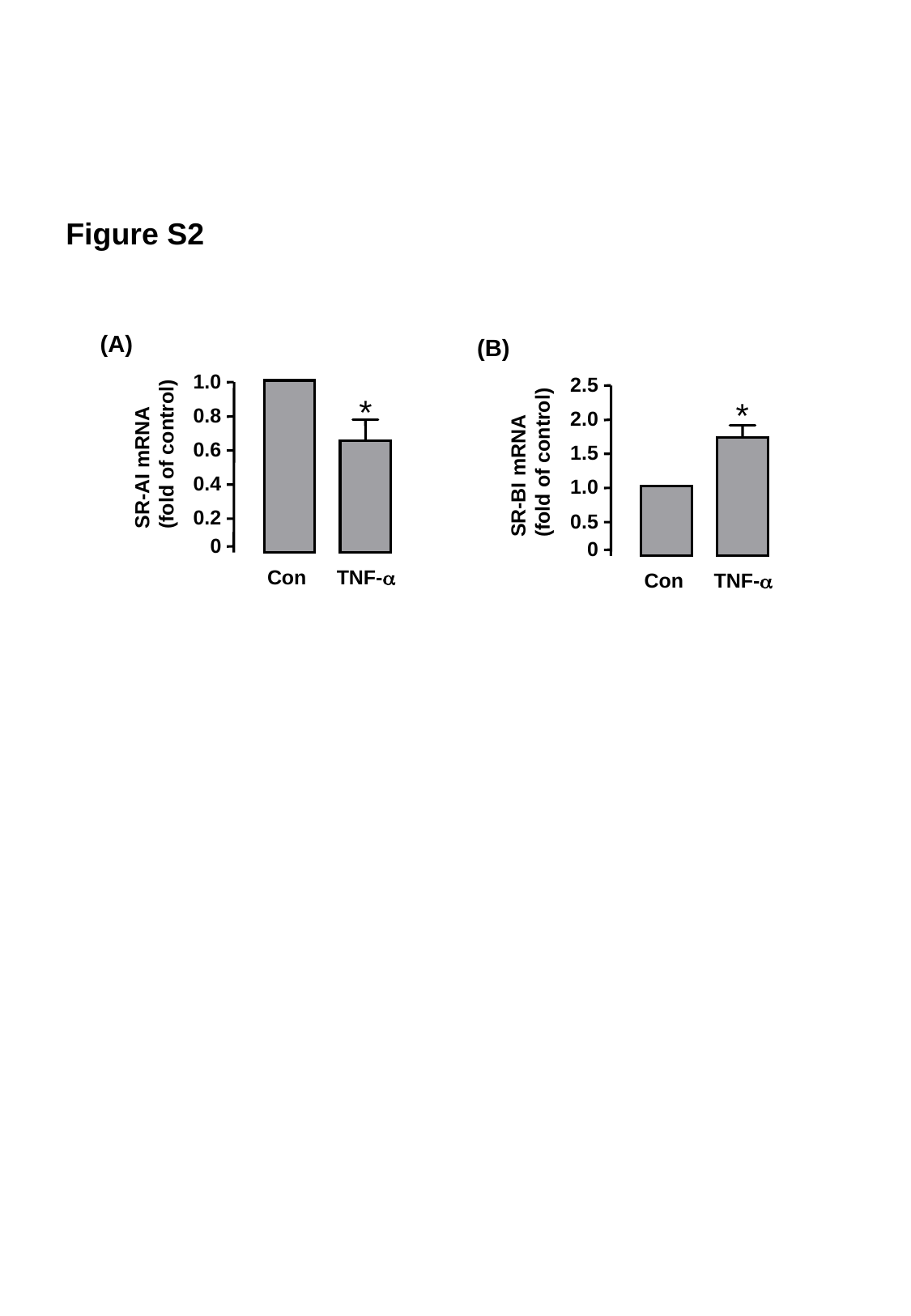

Figure S2
(A)
(B)
1.0
2.5
*
*
0.8
2.0
SR-AI mRNA
(fold of control)
SR-BI mRNA
(fold of control)
0.6
1.5
0.4
1.0
0.2
0.5
0
0
Con
TNF-α
Con
TNF-α

Supplement: Figure S2 — Effects of TNF-α (20 ng/ml) on SR-AI and SR-BI expression in THP-1 cells. The results are expressed as fold of control (Con). * P <0.05 vs Con, unpaired t -test, n = 3. (PPT) [file pone.0087059.s002.ppt]

## Slide 1
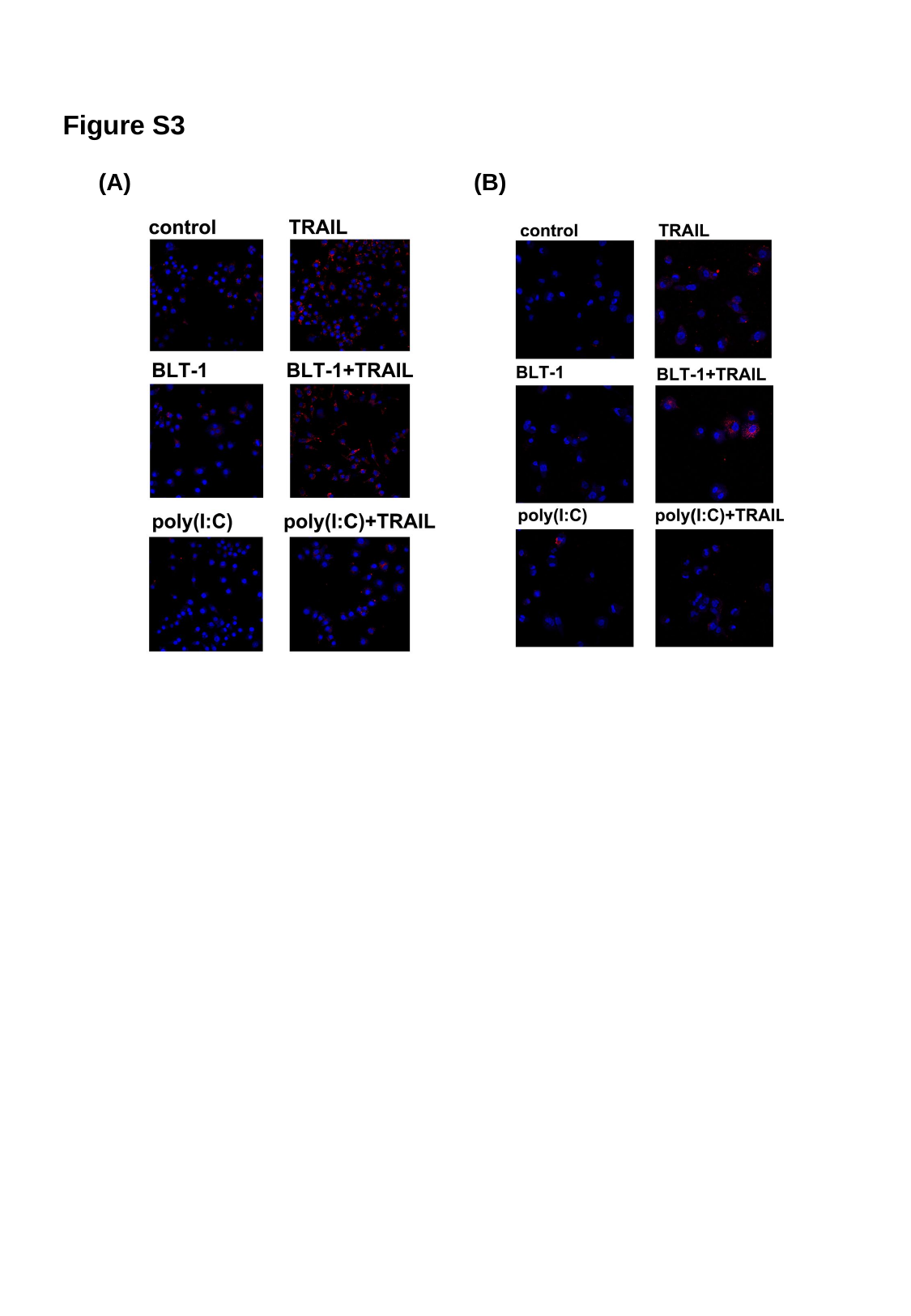

Figure S3
(A)
(B)

Supplement: Figure S3 — Effects of poly(I:C) and BLT-1 on rTRAIL-stimulated DiI-Ac-LDL uptake. Fluorescence microscopy images showing the effects of the SR-AI inhibitor poly(I:C) (1 µM) and the SR-BI inhibitor BLT-1 (5 µM) on rTRAIL-stimulated DiI-Ac-LDL uptake in RAW264.7 cells (A) and THP-1 cells (B). (PPT) [file pone.0087059.s003.ppt]
